# Retinoic Acid Signaling Plays a Restrictive Role in Zebrafish Primitive Myelopoiesis

**DOI:** 10.1371/journal.pone.0030865

**Published:** 2012-02-17

**Authors:** Dong Liang, Wenshuang Jia, Jingyun Li, Kui Li, Qingshun Zhao

**Affiliations:** 1 Model Animal Research Center, MOE Key Laboratory of Model Animal for Disease Study, Nanjing University, Nanjing, China; 2 Zhejiang Provincial Key Lab for Technology and Application of Model Organisms, School of Life Sciences, Wenzhou Medical College, Wenzhou, China; Hong Kong University of Science and Technology, China

## Abstract

Retinoic acid (RA) is known to regulate definitive myelopoiesis but its role in vertebrate primitive myelopoiesis remains unclear. Here we report that zebrafish primitive myelopoiesis is restricted by RA in a dose dependent manner mainly before 11 hpf (hours post fertilization) when anterior hemangioblasts are initiated to form. RA treatment significantly reduces expressions of anterior hemangioblast markers *scl*, *lmo2*, *gata2* and *etsrp* in the rostral end of ALPM (anterior lateral plate mesoderm) of the embryos. The result indicates that RA restricts primitive myelopoiesis by suppressing formation of anterior hemangioblasts. Analyses of ALPM formation suggest that the defective primitive myelopoiesis resulting from RA treatment before late gastrulation may be secondary to global loss of cells for ALPM fate whereas the developmental defect resulting from RA treatment during 10–11 hpf should be due to ALPM patterning shift. Overexpressions of *scl* and *lmo2* partially rescue the block of primitive myelopoiesis in the embryos treated with 250 nM RA during 10–11 hpf, suggesting RA acts upstream of *scl* to control primitive myelopoiesis. However, the RA treatment blocks the increased primitive myelopoiesis caused by overexpressing *gata4/6* whereas the abolished primitive myelopoiesis in *gata4/5/6* depleted embryos is well rescued by 4-diethylamino-benzaldehyde, a retinal dehydrogenase inhibitor, or partially rescued by knocking down *aldh1a2*, the major retinal dehydrogenase gene that is responsible for RA synthesis during early development. Consistently, overexpressing *gata4/6* inhibits *aldh1a2* expression whereas depleting *gata4/5/6* increases *aldh1a2* expression. The results reveal that RA signaling acts downstream of *gata4/5/6* to control primitive myelopoiesis. But, 4-diethylamino-benzaldehyde fails to rescue the defective primitive myelopoiesis in either *cloche* embryos or *lycat* morphants. Taken together, our results demonstrate that RA signaling restricts zebrafish primitive myelopoiesis through acting downstream of *gata4/5/6*, upstream of, or parallel to, *cloche*, and upstream of *scl*.

## Introduction

Vertebrate hematopoiesis arises successively in primitive and definitive waves at anatomically distinct sites during development [1]. Zebrafish primitive hematopoiesis occurs in the rostral end of anterior lateral plate mesoderm (ALPM) that is also known as rostral blood island (RBI), and the intermediate cell mass (ICM) that is an analog to extraembryonic yolk sac blood islands of mammals and birds [2]. RBI produces myeloid cells, head endothelium and endocardium whereas ICM, derived from posterior lateral plate mesoderm (PLPM), gives rise to erythrocytes, endothelial linage and a few myeloid cells [2], [3]. Zebrafish definitive hematopoiesis begins within the ventral wall of dorsal aorta in an aorta-gonad-mesonephros region at about 31 hpf (hour post fertilization). The precursors later colonize into kidney marrow (the equivalent of avian and mammalian bone marrow), thymus and pancreas, in which hematopoietic stem cells differentiate into erythroid, myeloid, and lymphoid lineages for lifetime [2], [3], [4].

Zebrafish primitive myelopoiesis is initiated in RBI to form anterior hemangioblasts marked by expressing transcription factors *scl*, *lmo2*, *gata2* and *etsrp* between 3- to 5-somite stages [2], [3], [5]. With development, a subset of the *scl* ^+^ precursors acquire myeloid cell fates by expressing myeloid-specific transcription factor *pu.1* (also known as *spi1*) [6], [7]. During 11- to 15-somite stages, the *pu.1^+^* precursors migrate toward head midline. They partially converge between eye and heart primordium and then abruptly migrate laterally, scattering into single cells across yolk sac [7], [8], [9]. After 18-somite stage, the precursors decline their *pu.1* expression but express mature myeloid-markers such as *lcp1* (lymphocyte cytosolic plastin 1, also known as *l-plastin*) identifying all myeloid cells including macrophages and neutrophils, *csf1r* (colony stimulating factor 1 receptor, also known as *fms*) marking macrophages and *mpx* (myeloperoxidase, also known as *mpo*) labeling neutrophils, respectively [6], [7], [8], [10]. It is believed that some macrophages later migrate posteriorly toward ICM whereas the myeloid precursors originated in ICM give rise to at least some granulocytes there [11]. The myeloid cells eventually develop into functional cells as early as 26 hpf [8].

Retinoic acid (RA), a derivative of vitamin A, not only plays crucial roles in vertebrate early development but also influences growth and differentiation of different adult cell types [12]. Since all-trans RA was introduced to treat human acute promyelocytic leukemia (APL) through promoting terminal granulocytic differentiation of malignant promyelocytes in the late 1980s [13], a number of studies have been performed to assess roles of RA signaling in regulating normal myelopoiesis [14], [15]. *In vitro* cell culture studies on the hematopoietic precursor cells derived from normal bone marrow or fetal liver suggest that RA generally enhances growth and differentiation of granulocyte progenitors [15] but may have different effects on hematopoietic cells depending on their maturational states [15], [16]. Analyses of the female SENCAR mice fed a vitamin A-deficient (VAD) diet reveal that shortage of RA signaling causes a dramatic expansion of myeloid cells in bone marrow, spleen and peripheral blood whereas addition of RA to the VAD diet completely reverses the abnormality [17]. The *in vivo* results suggest that the myeloid cell expansion is a direct result of RA deficiency. Consistent with the VAD mice, both cellular retinol-binding protein type I (*CRBPI*) knockout mice fed a VAD diet and the mice treated with RA receptor (RAR) antagonist BMS493 display a similar expansion of myeloid cells associated with an increase in immature granulocytes [18].

Although a lot of researches have been performed on analyzing roles of RA signaling in definitive myelopoiesis and differentiation of myeloid cells, its roles in primitive myelopoiesis remain unknown. In this study, we report that excessive RA suppresses zebrafish primitive myelopoiesis by restricting formation of anterior hemangioblasts in a dose dependent way. The defective myelopoiesis resulting from RA treatment before the end of gastrulation may be secondary to global loss of cells for ALPM fate whereas the defect resulting from RA treatment during 10–11 hpf should be ascribed to ALPM patterning shift. Performing analyses of epistatic relationships between RA signaling and the genes controlling zebrafish primitive myelopoiesis, we demonstrate that RA signaling acts downstream of *gata4/5/6*, upstream of, or parallel to, *cloche*, and upstream of *scl* to restrict zebrafish primitive myelopoiesis.

## Results

### RA inhibits zebrafish primitive myelopoiesis in a dose dependent way

**Figure 1:**
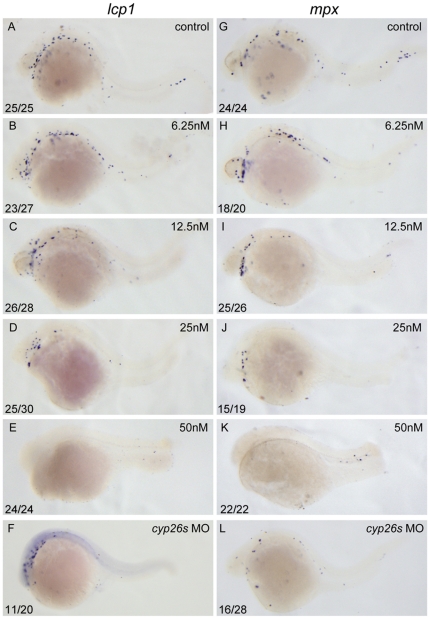
Excessive RA inhibits primitive myelopoiesis in zebrafish embryos in a dose dependent manner. All embryos are positioned anterior left and lateral front. Embryos were treated with vehicle DMSO (A, G), 6.25 nM (B, H), 12.5 nM (C, I), 25 nM (D, J) and 50 nM RA (E, K) respectively from 1–2-cell stage until 26 hpf or microinjected with *cyp26a1*-MO, *cyp26b1*-MO and *cyp26c1*-MO together at 1–2-cell stage (F, L). They were then examined for expressions of myeloid markers *lcp1* (A–F) and *mpx* (G–L) at 26 hpf by whole mount *in situ* hybridization. The number shown in the lower left-hand corner of each panel is the number of embryos exhibiting the typical phenotype shown in the panel to the number of embryos totally observed.

To examine roles of RA signaling in primitive myelopoiesis, we treated zebrafish embryos with RA at concentrations of 6.25, 12.5, 25 and 50 nM from 1–2-cell stage through 26 hpf, respectively. We then analyzed their expressions of myeloid cell markers *lcp1* and *mpx* at 26 hpf [6], [8], [10]. In control embryos, cells expressing *lcp1* and *mpx* were found to disperse throughout embryos (Figure 1A and 1G). The number of cells expressing *lcp1* and *mpx* was slightly reduced in the embryos treated with 6.25 nM RA (Figure 1B and 1H), significantly reduced in the embryos treated with 12.5 nM RA (Figure 1C and 1I) and 25 nM RA (Figure 1D and 1J), and almost completely abolished in the embryos treated with 50 nM RA (Figure 1E and 1K). The results demonstrate that exogenous RA inhibits zebrafish primitive myelopoiesis dose-dependently.

To examine whether elevation of endogenous RA levels by depleting Cyp26s, the enzymes responsible for metabolizing RA *in vivo* [12], limits zebrafish primitive myelopoiesis, we examined expressions of *lcp1* and *mpx* in *cyp26s* morphants at 26 hpf. When embryos were microinjected with control morpholino (MO), they displayed normal expressions of *lcp1* and *mpx* (data not shown). However, the embryos microinjected with *cyp26s*-specific MOs exhibited significantly reduced number of cells expressing *lcp1* and *mpx* (Figure 1F and 1L). Quantitative analysis by real-time PCR confirmed that expression levels of both *lcp1* and *mpx* in *cyp26s* morphants were significantly lower than (P<0.05) those in control embryos (Figure S1). The results reveal that *cyp26s* involve the maintenance of normal primitive myelopoiesis in zebrafish.

### RA plays its repressive role in zebrafish primitive myelopoiesis mainly before 11 hpf

**Figure 2:**
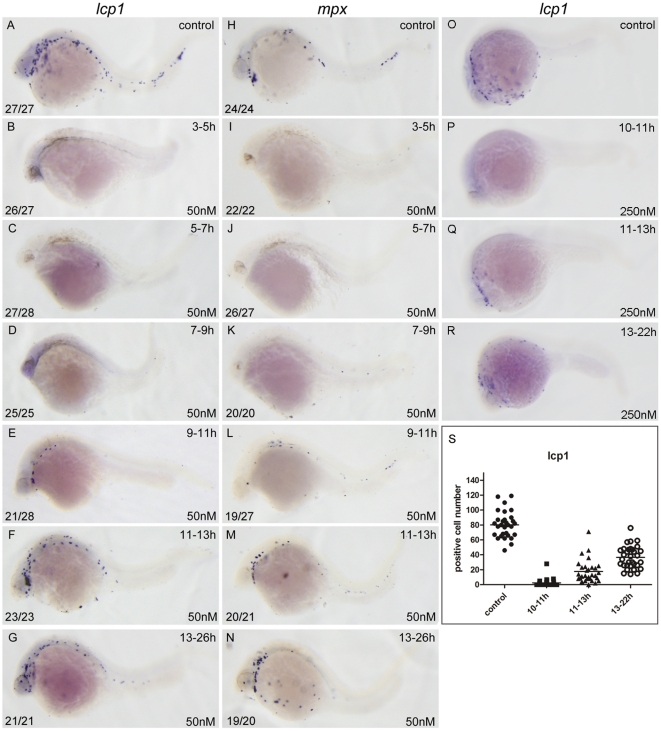
RA restricts the primitive myelopoiesis mainly before 11 hpf. All embryos are positioned anterior left and lateral front. Embryos were treated with vehicle DMSO (A, H, O) or with 50 nM RA (B–G, I–N) from 3 to 5 (B, I), 5 to 7 (C, J), 7 to 9 (D, K), 9 to 11 (E, L), 11 to 13 (F, M) and 13 to 26 hpf (G, N), or with 250 nM RA form 10 to 11 (P), 11 to 13 (Q), 13 to 22 hpf (R), respectively. They were then examined for expressions of myeloid markers *lcp1* (A–G, O–R) and *mpx* (H–N) at 26 hpf (A–N) or 22 hpf (O–R) by whole mount *in situ* hybridization. The number shown in the lower left-hand corner of each panel is the number of embryos exhibiting the typical phenotype shown in the panel to the number of embryos totally observed. The typical embryos expressing *lcp1^+^* cells at 22 hpf were shown in O–R. The scatter plot (S) shows the number of *lcp1^+^* cells counted from each of the embryos at 22 hpf with different treatment (control; 10–11 hpf RA treatment; 11–13 hpf RA treatment and 13–22 hpf RA treatment).

To determine when RA plays its repressive roles in zebrafish primitive myelopoiesis, we examined expressions of myeloid markers *lcp1* and *mpx* in the embryos treated with 50 nM RA from 3 to 5, 5 to 7, 7 to 9, 9 to 11, 11 to 13, and 13 to 26 hpf, respectively. The results showed that almost no expression of *lcp1* and *mpx* were found in the 26 hpf embryos treated with RA for 2 h during 3–5, 5–7 and 7–9 hpf (Figure 2B–D and 2I–K), and only a few myeloid cells expressing *lcp1* and *mpx* (Figure 2E and 2L) were found in the embryos treated with RA during 9–11 hpf. However, less affected expressions of *lcp1* and *mpx* were found in the embryos treated with RA during 11–13 hpf (Figure 2F and 2M) and nearly normal primitive myelopoiesis were found in the embryos treated with RA during 13–26 hpf (Figure 2G and 2N). The results suggest that RA signaling plays its repressive role in inhibiting zebrafish primitive myelopoiesis mainly at the developmental stages before 11 hpf.

Because RA restricts the primitive myelopoiesis in a dose dependent way (Figure 1), it is interesting to know whether the less affected and nearly normal primitive myelopoiesis in the zebrafish embryos treated with RA during 11–13 and 13–26 hpf respectively (Figure 2G and 2N) is due to the low amount of RA (50 nM). To test it, we analyzed the embryos treated with 5 times higher concentration (250 nM) of RA from 10 to 11, 11 to 13, and 13 to 22 hpf respectively. Counting the number of cells expressing myeloid markers *lcp1* in embryos at 22 hpf, we found the control embryos possessed 80.2±17.7 (n = 30) *lcp1^+^* cells (Figure 2O and 2S). The embryos treated with 250 nM RA for 1 h from 10 to 11 hpf only had 2.5±5.6 (n = 27) *lcp1^+^* cells (Figure 2P and 2S). The embryos treated with 250 nM RA for 2 h from 11 to 13 hpf held 17.7±15.2 (n = 30) *lcp1^+^* cells (Figure 2Q and 2S). The embryos treated with 250 nM RA from 13 to 22 hpf carried 36.7±14.9 (n = 32) *lcp1^+^* cells (Figure 2R and 2S). Though they exhibited significantly reduced number of *lcp1^+^* cells compared with control embryos, the embryos treated with 250 nM RA from 11 to 13 hpf or 13 to 22 hpf had much more *lcp1^+^* cells than the embryos treated with 250 nM RA for only 1 h from 10 to 11 hpf (Figure 2S). Similar results were observed when embryos were treated with 1000 nM RA (data not shown). Taken together, the results demonstrate that RA plays its repressive roles in zebrafish primitive myelopoiesis mainly at the developmental stage before 11 hpf and treating embryos with high concentration of RA (250 nM) during 10–11 hpf almost abolishes the primitive myelopoiesis.

### RA inhibits zebrafish primitive myelopoiesis by repressing formation of anterior hemangioblasts

**Figure 3:**
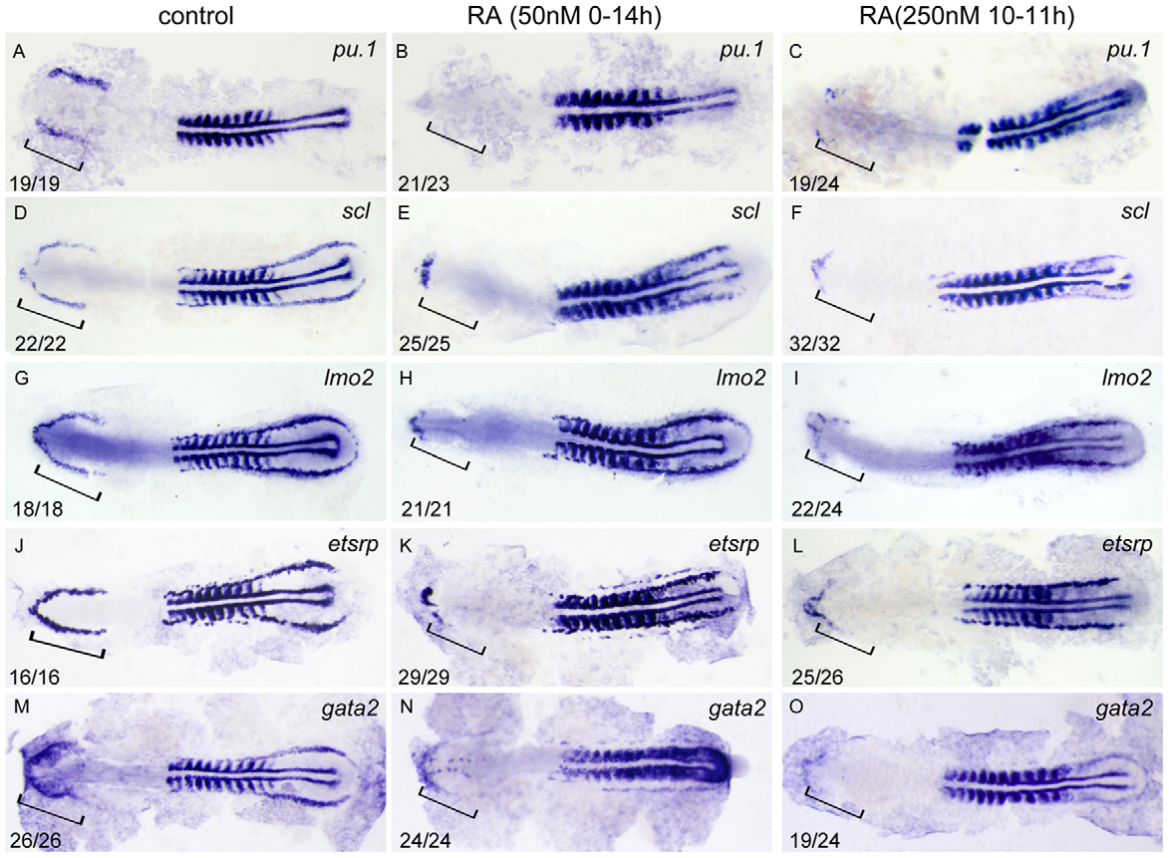
RA restricts the formation of anterior hemangioblasts in zebrafish embryos. All flat-mounted embryos are positioned anterior left and dorsal front. Embryos were treated with vehicle DMSO (A, D, G, J, M), 50 nM RA from 1–2-cell stage to 14 hpf (B, E, H, K, N) or 250 nM RA from 10 to 11 hpf (C, F, I, L, O), respectively. They were then examined for expressions of *pu.1* (A–C), *scl* (D–F), *lmo2* (G–I), *etsrp* (J–L), and *gata2* (M–O) in the rostral end of ALPM at 14 hpf by whole mount in *situ* hybridization. Expression of *myoD* in somites is used for staging. Bracket indicates the location of RBI. The number shown in the lower left-hand corner of each panel is the number of embryos exhibiting the typical phenotype shown in the panel to the number of embryos totally observed.

Zebrafish primitive myelopoiesis is initiated and specified in RBI during 11–14 hpf [3], [5]. To explore how RA acts to restrict primitive myelopoiesis, we first detected myeloid precursor maker *pu.1* expression in 14 hpf embryos treated with 50 nM RA from 1–2-cell stage to 14 hpf or 250 nM RA from 10 to 11 hpf, respectively. The results showed that expression of *pu.1* at 14 hpf was almost completely abolished in RBI of all the embryos treated with RA in two different ways (Figure 3B and 3C). Next, we found expressions of anterior hemangioblast markers *scl*, *lmo2*, *etsrp* and *gata2* were all significantly inhibited in RBI of the RA-treated embryos at 14 hpf (Figure 3D–O). The results suggest excessive RA represses formation of anterior hemangioblasts, resulting in developmental loss of myeloid cells.

### The inhibited formation of anterior hemangioblasts by excessive RA treatment from 1–2-cell to 14 hpf might be secondary to global loss of ALPM fate whereas the developmental defect by excessive RA treatment during 10–11 hpf should be due to ALPM patterning shift

**Figure 4:**
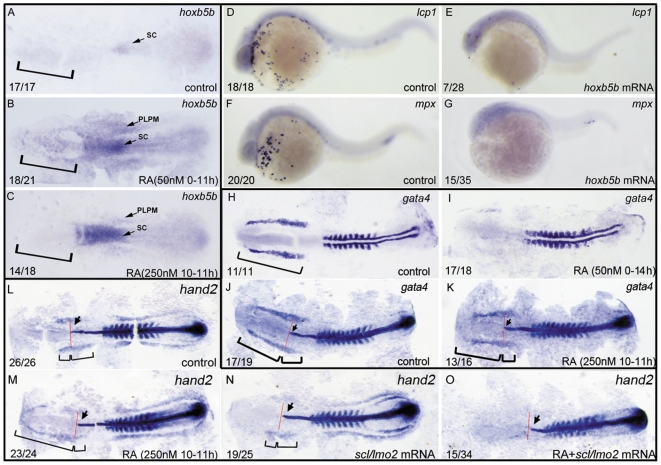
ALPM is lost in the embryos treated with 50 nM RA from 1–2-cell stage to 11 hpf but not eliminated in the ones treated with 250 nM RA during 10–11 hpf. All embryos including flat-mounted embryos (A–C, H–O) and whole-mounted embryos (D–G) are positioned anterior left and dorsal front (A–C, H–O) or lateral front (D–G). Embryos treated with vehicle DMSO (A), 50 nM RA from 1–2-cell stage to 11 hpf (B), and 250 nM RA during 10–11 hpf (C) were examined for *hoxb5b* expression at 11 hpf (A–C). Embryos treated with 50 nM RA treatment from 1–2-cell stage to 11 hpf displayed ectopically expression of *hoxb5b* in ALPM (B) but the ones treated with 250 nM RA during 10–11 hpf did not exhibit this ectopical expression (C). Compared with control embryos (D, F), overexpressions of *hoxb5b* by microinjecting embryos at 1–2-cell stage with *hoxb5b* mRNA significantly suppressed expressions of *lcp1* (E) and *mpx* (G) at 24 hpf. Embryos treated with vehicle DMSO (H, J), 50 nM RA from 1–2-cell stage to 14 hpf (I) or 250 nM RA during 10–11 hpf (K) were examined for expressions of ALPM marker *gata4* at 14 hpf. The location of ALPM at 11 hpf (A–C) or at 14 hpf (H, J, K) is indicated by bracket. Embryos treated with vehicle DMSO (L), 250 nM RA during 10–11 hpf (M, O), or microinjected with *scl*/*lmo2* mRNA (N, O) were examined for expressions of cardiac marker *hand2* at 14 hpf. Expression of *myoD* in somites (H–O) was used for staging and *ntl* expression was used for labeling embryonic axial mesoderm (J–O). The length between the anterior end of *gata4* expression domain (J, K) or *hand 2* expression domain (L–N) and the anterior end of *ntl* expression domain and the length between the posterior end of *gata4* expression domain (J, K) or *hand 2* expression domain (L–N) and the anterior end of *ntl* expression domain are marked by two different brackets. Red line denotes the anterior level of *ntl* expression domain (J–O). Arrow indicates the most anterior end of notochord marked by expression of *ntl* (J–O). sc: spinal cord; PLPM: posterior lateral plate mesoderm. The number shown in the lower left-hand corner of each panel is the number of embryos exhibiting the typical phenotype shown in the panel to the number of embryos totally observed.

Because RA is a posteriorizing factor for patterning anterior–posterior axis of embryos and excessive RA causes anterior truncation of embryos [12], [19], the defect of anterior hemangioblast formation from excessive RA treatment from 1–2-cell to 14 hpf could be due to global loss of ALPM tissue fates. To test this hypothesis, we first examined *hoxb5b* expression in the embryos treated with 50 nM RA from 1–2-cell to 11 hpf. Hoxb5b is a transcription factor that acts downstream of RA signaling in the forelimb field to restrict heart field potential in zebrafish embryos [20]. Normally, it is not expressed in LPM yet but in spinal cord at 11 hpf (Figure 4A) [20]. When embryos were treated with 50 nM RA from 1–2-cell stage to 11 hpf, we found *hoxb5b* was strongly induced to anteriorly expand its expression in spinal cord and ectopically express in LPM, especially in the presumptive ALPM of the RA-treated embryos (Figure 4B). Consistently, 25% (7/28) and 43% (15/35) of the embryos overexpressed with *hoxb5b* exhibited significantly reduced expressions of *lcp1* and *mpx* at 24 hpf, respectively (Figure 4D–G); however, knocking down *hoxb5b* did not rescue the suppressed primitive myelopoiesis in the RA-treated embryos (data not shown). Next, we examined *gata4* expression in the RA-treated embryos. Gata4 is a transcription factor that is normally expressed in ALPM of zebrafish embryos during anterior hemangioblast specification [21]. Treated with 50 nM RA from 1–2-cell stage to 14 hpf, the embryos exhibited almost abolished expression of *gata4* at 14 hpf (Figure 4I). The results are consistent with the idea that the inhibited formation of anterior hemangioblast resulting from excessive RA treatment from 1–2-cell stage to 14 hpf might be ascribed to global loss of cells for ALPM fate.

To explore direct roles of RA signaling in zebrafish primitive myelopoiesis, we have to exclude the global effect of excessive RA on embryonic development. We therefore analyzed the embryos treated with 250 nM RA for 1 h from 10 hpf to 11 hpf. As shown in Figure 4C, *hoxb5b* did not expand its expression to ALPM though it was indeed induced to expand its expression in spinal cord and ectopically express in PLPM of the RA-treated embryos. Consistently, the RA-treated embryos displayed a slightly reduced *gata4* expression in the region between the anterior end of its expression domain and the anterior end of *ntl* expression domain (marker for axial mesoderm) (Figure 4K) compared to control embryos (Figure 4J) whereas they had a significantly reduced expression in the region between the posterior end of its expression domain and the anterior end of *ntl* expression domain (Figure 4K) compared to control embryos (Figure 4J). Because the rostral part of ALPM is responsible for the formation of anterior hemangioblasts and its caudal part is responsible for the formation of cardiac precursors [21], the results strongly suggest that treatment with RA during 10–11 hpf does not abolish the anterior mesoderm of ALPM.

Previously, it was identified that embryos with inhibited anterior hemangioblast specification exhibited ectopic cardiac progenitors in the rostral portion of ALPM whereas overexpression of hemangioblast master regulators suppressed the cardiac development [22]. Because the 250 nM RA treatment during 10–11 hpf inhibited formation of anterior hemangioblasts (Figure 3C, 3F, 3I, 3L, 3O), we therefore asked whether the RA treatment skewed the developmental potential of the rostral tissues into caudal fates. To answer the question, we examined expression of *hand2*, the heart field marker [22], [23], in the RA-treated embryos. In normal embryos, *hand2* was confined to the caudal region of ALPM and whole PLPM (Figure 4L). Treated with 250 nM RA during 10–11 hpf, the embryos exhibited greatly reduced expression of *hand2* in caudal portion of ALPM but ectopical expression of *hand2* in the whole rostral portion of ALPM (Figure 4M). Overexpressed with *scl* and *lmo2* (master regulators of hemangioblasts), the control embryos displayed significantly reduced expression of *hand2* in the caudal portion of ALPM (Figure 4N) whereas the RA-treated embryos showed greatly reduced (15/34) or even completely lost (19/34) expression of *hand2* not only in the rostral portion but also in the caudal portion of ALPM (Figure 4O). The results suggest that RA treatment during 10–11 hpf shifts ALPM patterning by skewing the developmental potential of rostral tissues into the caudal fate.

### RA signaling acts upstream of *scl/lmo2* to control zebrafish primitive myelopoiesis

**Figure 5:**
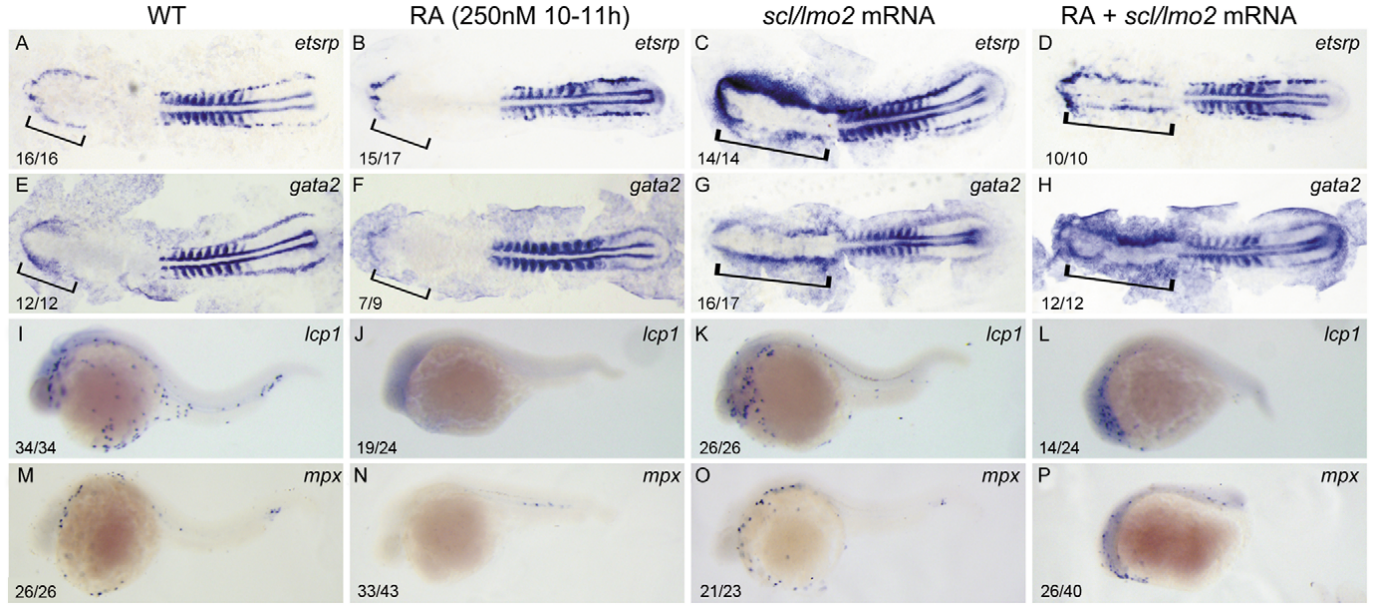
Overexpressions of *scl* and *lmo2* into RA-treated zebrafish embryos partially rescue the defective primitive myelopoiesis. Both flat-mounted embryos (A–H) and whole-mounted embryos (I–P) are positioned anterior left and dorsal front (A–H) or lateral front (I–P). Embryos were treated with vehicle DMSO (A, E, I, M), 250 nM RA during 10 to 11 hpf (B, F, J, N), or microinjected with *scl* and *lmo2* mRNA at 1–2-cell stage (C, G, K, O), or microinjected with *scl* and *lmo2* mRNA at 1–2-cell stage and then treated with 250 nM RA during 10 to 11 hpf (D, H, L, P), respectively. They were then examined for expressions of hemangioblast markers *etsrp* (A–D) and *gata2* (E–H) at 14 hpf, and myeloid markers *lcp1* (I–L) and *mpx* (M–P) at 24 hpf by whole mount in *situ* hybridization. Expression of *myoD* in somites was used for staging (A–H). Bracket indicates the location of RBI (A, B, E, F), and ALPM (C, D, G, H). The number shown in the lower left-hand corner of each panel is the number of embryos exhibiting the typical phenotype shown in the panel to the number of embryos totally observed.

*scl* and *lmo2* are known the two key master regulators for regulating anterior hemangioblasts [22]. Because the 250 nM RA treatment during 10–11 hpf inhibited expressions of anterior hemangioblasts master regulators *scl* and *lmo2* (Figure 3C, 3F, 3I, 3L, and 3O), we hypothesized that RA signaling acts upstream of *scl*/*lmo2* to control zebrafish primitive myelopoiesis. To confirm this hypothesis, we performed rescue experiments by overexpressing *scl* and *lmo2* into the RA-treated embryos. The results showed that the overexpression not only rescued the suppressed expressions of both *etsrp* and *gata2* in the rostral part but also induced their ectopical expressions in the caudal part of ALPM (Figure 5B, 5D; 5F and 5H). Consequently, the inhibited expressions of both *lcp1* and *mpx* were partially rescued at 24 hpf when the RA-treated embryos were microinjected with *scl* and *lmo2* mRNA (Figure 5J, 5L, 5N and 5P). Interestingly, the control embryos overexpressed with *scl* and *lmo2* exhibited similar expression levels of *lcp1* and *mpx* to wild type embryos at 24 hpf (Figure 5I, 5K, 5M and 5O) though they had significantly up-regulated expressions of *etsrp* and *gata2* in ALPM at 14 hpf (Figure 5A, 5C, 5E and 5G). Consistently, our quantitative RT-PCR analysis of the expression levels of *lcp1* and *mpx* for the myeloid cells originated from RBI by removing the trunk and tail of the embryos with different treatments showed that overexpressions of *scl* and *lmo2* significantly up-regulated (P<0.01) the expressions of *lcp1* and *mpx* in the RA-treated embryos but did not change them (P>0.05) in control embryos (Figure S2).

Taken together, the results suggest that RA signaling acts upstream of *scl/lmo2* to control zebrafish primitive myelopoiesis.

### RA signaling acts upstream of, or parallel to, cloche to inhibit zebrafish primitive myelopoiesis

**Figure 6:**
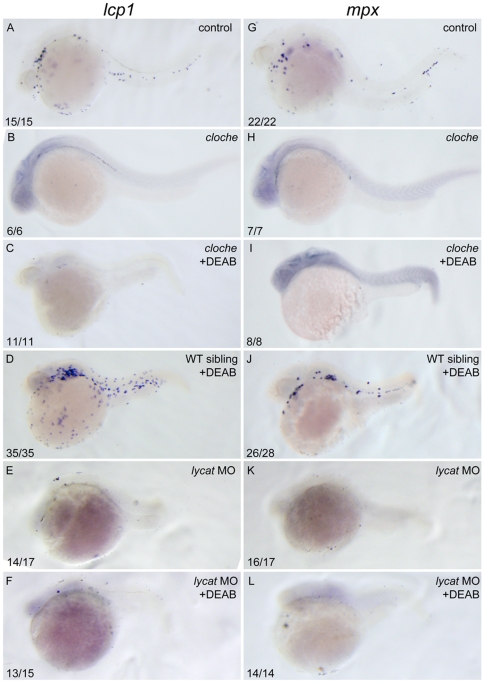
DEAB cannot rescue the defective primitive myelopoiesis in *cloche* or *lycat* knockdown embryos. All embryos are positioned anterior left and lateral front. Wild type siblings (A, G), *cloche* (B, H) and the embryos microinjected with *lycat*-MO at 1–2-cell stage (E, K) were treated with vehicle DMSO whereas *cloche* (C, I), *cloche* siblings (D, J) and *lycat*-MO knockdown (F, L) embryos were treated with 10 µM DEAB from 1–2-cell stage until 26 hpf. They were then examined for expressions of myeloid markers *lcp1* (A–F) and *mpx* (G–L) at 26 hpf by whole mount *in situ* hybridization. The number shown in the lower left-hand corner of each panel is the number of embryos exhibiting the typical phenotype shown in the panel to the number of embryos totally observed.

*cloche* acts upstream of *scl* to regulate development of hematopoietic and vascular tissues [24], [25], [26]. To explore epistatic relationship of RA signaling with *cloche*, we should examine expression changes of *cloche* in RA signaling-altered embryos. Unfortunately, we could not monitor *cloche* expressions because of low expression of the only candidate *lycat* [27]. We therefore examined expressions of *lcp1* and *mpx* in *cloche* embryos treated with 10 µM DEAB (4-diethylamino-benzaldehyde), a retinal dehydrogenase inhibitor, to see whether decreasing RA signaling could rescue the defect of primitive myelopoiesis in *cloche*. *cloche* embryos hardly expressed *lcp1* and *mpx* (Figure 6B and 6H) while their heterozygous siblings showed normal expression levels (Figure 6A and 6G). When treated with 10 µM DEAB, the sibling embryos of *cloche* exhibited significantly increased expressions of *lcp1* and *mpx* (Figure 6D and 6J), which is consistent with RA's suppressing role in zebrafish primitive myelopoiesis. However, DEAB treatment could not rescue expressions of the two genes in *cloche* embryos (Figure 6C and 6I). It is known that knocking down *lycat* phenocopies *cloche* [27]. We therefore examined the primitive myelopoiesis in *lycat* morphants that were treated with 10 µM DEAB. Similar to that in *cloche* embryos, DEAB treatment did not rescue expressions of *lcp1* and *mpx* that were depleted in *lycat* knockdown embryos (Figure 6E, 6F, 6K and 6L). Taken together, these results support that RA signaling lies upstream of, or parallel to, *cloche* to suppress the primitive myelopoiesis.

### RA signaling acts downstream of *gata4/5/6* to control zebrafish primitive myelopoiesis

*gata4*, *gata5* and *gata6* are known to lie above *cloche* and *scl* controlling primitive myelopoiesis [21]. Because *gata4* expression was slightly reduced in the 250 nM RA-treated embryos (Figure 4K), we therefore examined whether the expressions of *gata5* and *gata6* were changed in the embryos with the same treatment. When compared to control embryos (Figure S3A and S3C), the RA-treated embryos exhibited similar expression of *gata5* but somehow increased expression of *gata6* in the region between the anterior ends of their expression domains and the anterior end of *ntl* expression domain, respectively (Figure S3B and S3D); however, they displayed significantly increased *gata5* expression but greatly reduced *gata6* expression in the region between the posterior end of their expression domains and the anterior end of *ntl* expression domain, respectively (Figure S3). Because the rostral part of ALPM is responsible for the formation of anterior hemangioblasts [21], these results imply that RA signaling should not act upstream of *gata4/5/6* to control the primitive myelopoiesis.

**Figure 7:**
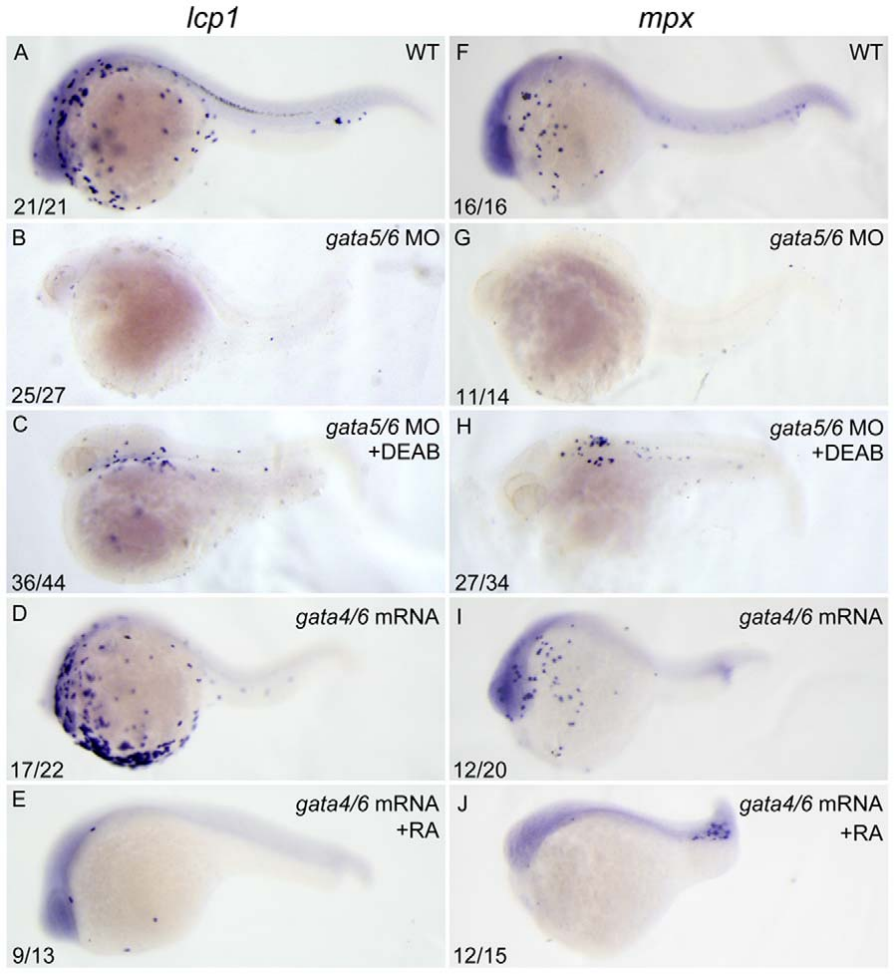
RA inhibits primitive myelopoiesis by acting downstream of *gata4/5/6*. All embryos are positioned anterior left and lateral front. Embryos were microinjected with *gata5*-MO and *gata6*-MO together (B, C, G, H) at 1–2-cell stage and then treated with 10 µM DEAB (C, H) or vehicle DMSO (B, G) immediately, or microinjected with *gata4* mRNA and *gata6* mRNA together (D, E, I, J) and then treated with 250 nM RA (E, J) or vehicle DMSO (D, I) from 10 to 11 hpf, respectively. They were then examined for expressions of myeloid markers *lcp1* (A–E) and *mpx* (F–J) at 24 hpf by whole mount *in situ* hybridization. The number shown in the lower left-hand corner of each panel is the number of embryos exhibiting the typical phenotype shown in the panel to the number of embryos totally observed.

To explore whether RA signaling works downstream of *gata4/5/6*, we first examined whether reducing RA signaling could reverse the phenotype of the abolished primitive myelopoiesis in *gata4/5/6* depleted embryos. Because *gata4* is not expressed in *gata5/6* double morphants, the double knockdown is actually a triple knockdown [21]. We therefore depleted expressions of *gata4/5/6* by microinjecting *gata5*-MO and *gata6*-MO into zebrafish embryos. When the *gata4/5/6* depleted embryos were treated with 10 µM DEAB from 1–2-cell stage to 24 hpf, the abolished expressions of *lcp1* and *mpx* (Figure 7B and 7G) were well rescued in 82% (36/44) and 79% (27/34) of the embryos respectively (Figure 7C and 7H). Additionally, treating the *gata4/5/6* depleted embryos with 10 µM DEAB starting from different developmental stages including 3 hpf, 5 hfp, 7 hpf and 9 hpf but not 11 hpf well rescued the ablated expressions of *lcp1* and *mpx* (Figure S4). The result suggests that RA signaling works downstream *gata4/5/6* to inhibit the primitive myelopoiesis. To further support this conclusion, we performed overexpression experiment. We found that overexpressions of *gata4* and *gata6* in control embryos significantly increased expressions of *lcp1* and *mpx* (Figure 7D and 7I). However, the increased expressions of *lcp1* and *mpx* were greatly reduced when the embryos were treated with 250 nM RA during 10–11 hpf (Figure 7E and 7J). Quantitative analyses of the embryos with different treatments by real-time RT-PCR confirmed that RA treatment blocked the increased expressions of *lcp1* and *mpx* in the embryos overexpressed with *gata4/6* mRNAs (Figure S5). Taken together, the results demonstrate that RA signaling works downstream of *gata4/5/6* to control zebrafish primitive myelopoiesis.

**Figure 8:**
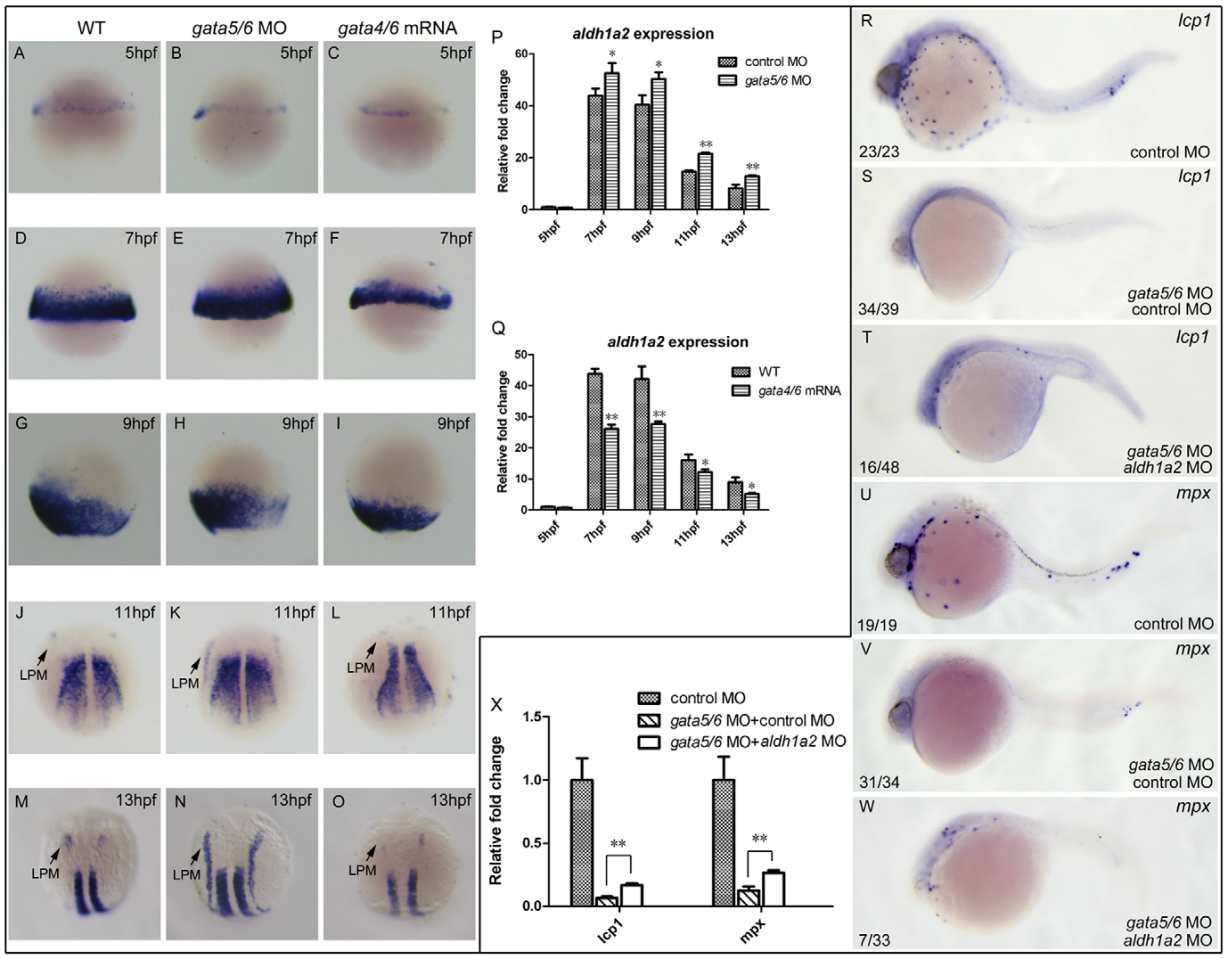
*aldh1a2* is one of downstream target genes of *gata4/5/6* to affect zebrafish primitive myelopoiesis. Embryos are positioned animal pole top and ventral front (A–I), anterior top and dorsal front (J–O), and anterior left and lateral front (R–W). Embryos were microinjected with control MO (A, D, G, J, M, R, U), *gata5*-MO plus *gata6*-MO (B, E, H, K, N), *gata4* mRNA plus *gata6* mRNA (C, F, I, L, O), *gata5*-MO and *gata6*-MO plus control MO (S, V), *gata5*-MO and *gata6*-MO plus *aldh1a2* MO (T, W) at 1–2-cell stage, respectively. They were then examined for expressions of *aldh1a2* at 5 hpf (A–C), 7 hpf (D–F), 9 hpf (G–I), 11 hpf (J–L) and 13 hpf (M–O), *lcp1* (R–T) and *mpx* at 24 hpf (U–W) by whole mount *in situ* hybridization, respectively. qRT-PCR was performed to confirm the relative expression level changes of *aldh1a2* in *gata4/5/6* depleted embryos (P) or in *gata4/6* overexpressed embryos (Q) at 5, 7, 9, 11, 13 hpf, and those of *lcp1* and *mpx* at 24 hpf (X). The number shown in the lower left-hand corner of each panel (R–W) is the number of embryos exhibiting the typical phenotype shown in the panel to the number of embryos totally observed. *: P<0.05; **: P<0.01.

*aldh1a2* is the major gene that is in charge of RA synthesis during zebrafish early development [28], [29]. To explore whether it is responsible for mediating *gata4/5/6* signaling to control the primitive myelopoiesis, we examined expression changes of *aldh1a2* in *gata4/5/6* depleted or *gata4/6* overexpressed embryos. The results showed that *aldh1a2* expression was not changed in the two types of embryos at 5 hpf (Figure 8A–C). It was slightly expanded in the ventral mesoderm of *gata4/5/6* depleted embryos but significantly decreased in *gata4/6* overexpressed embryos at 7 hpf and 9 hpf respectively (Figure 8D–I). At 11 hpf and 13 hpf, *aldh1a2* was significantly increased and ectopically expressed in LPM of *gata4/*5/6 depleted embryos whereas its expression was significantly reduced in both LPM and somites in *gata4/6* overexpressed embryos (Figure 8J–O). Quantitative analyses by real-time RT-PCR confirmed that its expression was significantly increased in *gata4/*5/6 depleted embryos (Figure 8P) but reduced in *gata4/6* overexpressed embryos (Figure 8Q) at 7, 9, 11 and 13 but not 5 hpf, respectively. Moreover, the abolished expressions of *lcp1* and *mpx* in *gata4/5/6* depleted embryos were partially rescued to express respectively when the *gata4/5/6* depleted embryos were microinjected with *aldh1a2*-MO (Figure 8R–W). Quantitative analysis by real-time PCR further confirmed this rescue result (Figure 8X). Taken together, our observations suggest *aldh1a2* is one of the candidate genes that work downstream of *gata4/5/6* to control the primitive myelopoiesis.

## Discussion

Unlike mammals and birds whose primitive hematopoiesis generates a small number of primitive macrophages and no granulocytes, zebrafish primitive hematopoiesis produces functional granulocytes and macrophages [2], [6], [8]. Although it is not known how zebrafish primitive myeloid pathway relates to mammalian definitive myelopoiesis, the primitive myelopoiesis has been widely used to assess myelopoiesis [11], [30]. RA has been implicated in regulating definitive myelopoiesis [14], [15], but little is known about its role in primitive myelopoiesis. Here we report that RA suppresses zebrafish primitive myelopoiesis in a dose dependent way (Figure 1) mainly before 11 hpf when anterior hemangioblasts are initiated to form (Figure 2) whereas reducing endogenous RA signaling increases the myelopoiesis (Figure 6D and 6J). Our results are consistent with the previous observations from VAD mice, *CRBPI*-knock-out mice and RAR antogonist BMS493 treated mice showing that shortage of RA signaling causes myeloid cell expansion [17], [18], and recently researches revealing that DEAB increases myeloid cells in zebrafish [31]. However, studies on APL treatment reveal that RA relieves the disease by promoting terminal granulocytic differentiation of malignant promyelocytes [12], [15]. Consistently, *in vitro* researches show that RA enhances differentiation of granulocyte progenitors derived from the hematopoietic precursor cells in bone marrow or fetal liver [14] and generation of differentiated hematopoietic cells from human embryonic stem cell-derived hemato-vascular precursors [32]. Taken together, these observations suggest that RA restricts normal myelopoiesis but promotes myeloid differentiation of hematopoietic precursors.

Zebrafish primitive myelopoiesis initiates in RBI by expressing transcription factors such as *scl*, *lmo2*, *gata2*, and *etsrp* [5], [24], [33] and then acquires myeloid cell fate through expressing *pu.1* [6], [7]. Recent reports have shown that treating embryos with excessive RA before late gastrulation reduces or eliminates the expression of ALPM markers including *gata4* [34], *scl* and *pu.1* [35]. Consistently, we report in this study that treating embryos with exogenous RA in a concentration as low as 50 nM before 11 hpf abolishes expressions of the ALPM markers including *gata4* (Figure 4I), *pu.1* (Figure 3C), *scl* (Figure 3F), *lmo2* (Figure 3I), *etsrp* (Figure 3L) and *gata2* (Figure 3O), leading to developmental loss of primitive myelopoiesis.

It is known that excessive RA posteriorizes vertebrate embryos during gastrulation [12], [19], raising the possibility that the developmental loss of ALPM markers from excessive RA treatment at early development might be secondary to global loss of cells for ALPM tissue fate. Consistent with the idea, our results showed that treating embryos with 50 nM RA from 1–2-cell stage to 11 hpf caused *hoxb5b*, an RA direct target gene that acts downstream of RA signaling in the forelimb field to restrict heart field potential in zebrafish embryos [20], expanding its expression to whole ALPM of 11 hpf embryos (Figure 4B). Additionally, *gata4*, an ALPM marker [21], was almost abolished its expression in whole ALPM of the 50 nM RA-treated embryos (Figure 4I), which supports the conclusion that excessive RA treatment at early stage may cause global loss of cells for ALPM tissue fate.

However, the induced expression of *hoxb5b* was not expanded to ALPM but confined to PLPM in the embryos treated with 250 nM RA during 10–11 hpf (Figure 4C). Consistently, the RA-treated embryos only slightly reduced expression of *gata4*, an ALPM marker [21], in the rostral portion of ALPM (Figure 4K). Moreover, overexpression of *scl* and *lmo2* partially rescued the primitive myelopoiesis abolished in the RA-treated embryos (Figure 5J, 5L, 5N and 5P) but it cannot increase the development of myeloid cells ectopically in wild type embryos [36] (also see Figure 5I, 5K, 5M and 5O). Taken together, these observations suggest the suppressed formation of anterior hemangioblasts in the 250 nM RA-treated embryos cannot be ascribed to the abolishment of anterior mesoderm in ALPM. Therefore, the embryos treated with 250 nM RA during 10–11 hpf provide us a useful model for analyzing direct roles of RA signaling in zebrafish primitive myelopoiesis.

The entire ALPM in early zebrafish embryo possesses cardiac developmental potential and the specification of anterior hemangioblasts represses formation of cardiac progenitors in the rostral portion of ALPM [22]. Embryos with defects in anterior hemangioblast specification exhibit ectopic cardiac progenitors in the rostral portion whereas overexpression of hemangioblast master regulators suppresses the cardiac development in the caudal portion of ALPM [22]. In this study, we report that *hand2*, the marker of cardiac precursors normally expressed in the caudal end of ALPM (Figure 4L) [22], [23], [37], was ectopically expressed in the whole rostral portion of ALPM in the RA-treated embryos that lacked anterior hemangioblasts (Figure 4M) whereas overexpressions of *scl* and *lmo2* greatly reduced or even eliminated the ectopic expression of the cardiac marker that was expressed in the RA-treated embryos (Figure 4M and 4O). The results demonstrate that RA signaling shifts the developmental potential of the anterior mesoderm into the posterior fate of ALPM, enabling the precursors in this region will no longer develop into myeloid cells.

Zebrafish primitive myelopoiesis is genetically controlled by many genes. *scl* plays a pivotal role in primitive hematopoiesis [3]. It works together with *lmo2* to define hemangioblast during zebrafish early development [38]. Epistatic analyses have shown that *cloche* acts upstream of *scl*, *lmo2*, and *etsrp* [5], [26], [33], [39], and *gata4/5/6* acts upstream of, or parallel to *cloche* and upstream of *scl* [21] to control zebrafish primitive myelopoiesis. Recently, RA was demonstrated to inhibit the commitment of mesodermal cells to hematopoietic fates by functioning downstream of *cdx4* and upstream of *scl* [35]. In this study, we report that RA suppresses the primitive myelopoiesis by inhibiting expressions of *scl* and *lmo2* (Figure 3F and 3I) and the inhibited primitive myelopoiesis is partially rescued when *scl* and *lmo2* are overexpressed (Figure 5L and 5P). The results demonstrate that RA signaling works upstream of *scl* to regulate the primitive myelopoiesis. However, the abolished primitive myelopoiesis in neither *cloche* embryos nor *lycat* morphants can be rescued by DEAB (Figure 6), indicating that RA works upstream of, or parallels to, *cloche*.

RA has been shown to enhance *GATA4* expression in F9 cells [40] and P19 cells [41], and improve both *GATA4* and *GATA6* but not *GATA5* expression in mouse embryonic stem cells (ES) [42], [43]. In VAD quail embryos, *GATA4* fails to be expressed whereas adding RA restores its expression pattern [44]. In *Xenopus* embryos, excessive RA causes expanded expressions of *GATA4/5/6* [45]. Consistent with its enhancing expressions of the GATA factors, RA commits mouse ES cells to differentiate into endoderm by enhancing expressions of *GATA6* and *GATA4* [42], [46], and directs urothelial specification of the ES cells by activating *GATA4/6* signaling [43]. However, *gata4* expression was significantly reduced or completely abolished respectively in zebrafish embryos at 8-somite stage that were treated with 100 nM RA or 300 nM RA at 40% epiboly for 1 h [34]. We show here that the embryos treated with 50 nM RA from 1–2-cell stage to 14 hpf displayed almost no *gata4* expression (Figure 4I) whereas the embryos treated with 250 nM RA during 10–11 hpf exhibited slightly reduced *gata4* expression (Figure 4K), unchanged *gata5* expression (Figure S3B) and somehow increased *gata6* expression (Figure S3D) in the rostral portion of their ALPM. Moreover, we revealed that DEAB could well rescue the abolished primitive myelopoiesis in *gata4/5/6* depleted embryos whereas RA blocked the increased primitive myelopoiesis in *gata4/6* overexpressed embryos (Figure 7). Taken together, our results demonstrate that RA signaling acts downstream of *gata4/5/6* to control zebrafish primitive myelopoiesis. Consistently, we found overexpressions of *gata4/6* significantly inhibited expression of *aldh1a2*, the major gene that is responsible for RA synthesis during early development [28], [29], whereas depleting *gata4/5/6* increased *aldh1a2* expression in zebrafish embryos (Figure 8). However, knocking down *aldh1a2* only partially rescued the abolished primitive myelopoiesis in *gata4/5/6* depleted embryos (Figure 8), which is consistent with recent survey revealing that knocking down both *aldh1a2* and *aldh1a3* cannot recapitulate the hematopoietic expansion like happening in DEAB-treated embryos [31]. The results suggest that other DEAB-sensitive aldehyde dehydrogenases should work downstream of *gata4/5/6* to restrict zebrafish primitive myelopoiesis.

In summary, our results demonstrate that RA signaling acts downstream of *gata4/5/6*, upstream of, or parallel to, *cloche*, and upstream of *scl* to suppress zebrafish primitive myelopoiesis by restricting the formation of anterior hemangioblasts (Figure S6) in a dose dependent way.

## Materials and Methods

### Ethics statement

Zebrafish used in this study are housed in the zebrafish facility of Model Animal Research Center (MARC), Nanjing University, in accordance with IACUC-approved protocol (MARC-AP#: QZ01).

### Pharmaceutical treatment of zebrafish embryos with RA and DEAB

All-trans RA, 4-diethylamino benzaldehyde (DEAB) and dimethyl sulfoxide (DMSO) were purchased from Sigma-Aldrich (MO, USA). A stock solution of 0.2 mM RA and 1 mM DEAB were prepared with DMSO and stored at –80°C. Embryos collected from wild type zebrafish or *cloche* mutant [39] were grown at 28.5°C and staged as previously reported [47]. Different concentrations of RA or DEAB were administrated to increase or reduce RA signaling in zebrafish embryos, respectively. The final concentration of DMSO in each treatment and control is 0.1%.

### Microinjection of morpholinos into zebrafish embryos

Morpholinos (MOs) were purchased from Gene Tools (http://www.gene-tools.com). The sequences of antisense MOs were listed in Table S1. Control MO was the standard MO provided by Gene Tools. MOs were dissolved in nanopure water and injected into the embryos at 1–2-cell stages. The injected amount per embryo was about 1 nl solution containing 1 ng *cyp26a1*-MO plus 2 ng *cyp26b1*-MO plus 2 ng *cyp26c1*-MO, 8 ng *aldh1a2*-MO plus 6.25 ng *gata5*-MO plus 1.25 ng *gata6*-MO, 6.25 ng *gata5*-MO plus 1.25 ng *gata6*-MO, or 4 ng *lycat*-MO, respectively.

### In vitro synthesis of mRNA and microinjection of mRNAs into zebrafish embryos

To synthesize mRNA *in vitro*, we first cloned the full-length coding sequences of *gata4* [48], *gata6* [49], *hoxb5b* [34], *lmo2* [24] and *scl* [33] by RT-PCR using primers listed in Table S2. The cDNAs were cloned into pGEM-T vector (Promega, USA) using HF PCR Kit (Takara, Japan) and then confirmed their identities by direct sequencing from both ends. The fragments were then cloned into the modified expression vector of pxT7 with 5′- and 3′-UTR of zebrafish *β-globin* [50] by digesting with *EcoR*I. The capped and tailed mRNA of these genes was generated from linearized pxT7 plasmid containing zebrafish *gata4*, *gata6*, *hoxb5b*, *lmo2* or *scl* coding sequence using the mMESSAGE mMACHINE T7 Ultra Kit (Ambion, USA). The synthesized mRNAs were further purified with RNeasy Kit (Qiagen, German) to remove the free nucleotides. Embryos were microinjected at 1–2-cell stage. The injected amount per embryo was about 1 nl solution containing 100 pg of *scl* and *lmo2*, 150 pg of *hoxb5b*, or 25 pg of *gata4* and *gata6*, respectively.

### Whole mount in situ hybridizations

Whole mount *in situ* hybridizations were performed as described previously [51]. The templates for anti-sense RNA probes including *etsrp* [52], *gata2* [24], *gata5* [53], and *hand2* [23] were cloned using the primers listed in Table S3. The templates for anti-sense RNA probes including *scl*, *gata4*, *gata6* and *hoxb5b* are the same as those used for mRNA synthesis. The plasmids containing the probe templates of *mpx*, *lcp1*, *pu.1* [6], *ntl* and *lmo2* [24] were kindly given by Dr. Yeh [54] and Dr. Zhang [55]. Other probes including *myoD*, *cyp26a1* and *aldh1a2* were used as previously reported [19]. Photomicrographs were taken using Olympus DP71 digital camera (Olympus, Japan). Digital images were further processed for brightness and contrast with Adobe Photoshop software.

### Real-time RT-PCR assay

Quantitative reverse transcription-polymerase chain reaction (qRT-PCR) was performed to examine the relative expression levels of *aldh1a2*, *lcp1*, *mpx* and *β-actin* (for control) using the ABI Prism 7300 Sequence Detector (PE Biosystems, USA). Total RNAs were extracted from 30 embryos using Trizol reagent (Invitrogen, USA) for each assay. RNA was reverse-transcribed and PCR was performed using the SYBR Green method following the manufacturer's protocol. Primers used for qRT-PCR were listed in Table S4. Relative quantification of each gene expression was performed by the comparative CT methods. Transcript level of examined genes was normalized to *β-actin* mRNA level according to standard procedures. Data were shown as means ± SD (standard deviation). Comparisons between numerical data were evaluated by unpaired Student *t* test. P value less than 0.05 was considered statistically significant. Every experiment was independently performed at least twice.

## Supporting Information

Figure S1 **qRT-PCR analysis shows the relative expression levels of** ***lcp1*** **and** ***mpx*** **are significantly reduced in** ***cyp26s*** **morphants.** Embryos were microinjected with control MO (Control MO) or *cyp26a1*-MO plus *cyp26b1*-MO plus *cyp26c1*-MO (*cyp26s* MO) at 1–2-cell stage and then examined for expressions of myeloid markers *lcp1* and *mpx* at 26 hpf by qRT-PCR. *: P<0.05.
(TIF)

Figure S2 **qRT-PCR analysis shows overexpression of** ***scl*** **and** ***lmo2*** **into zebrafish embryos partially rescues the defective primitive myelopoiesis in the embryos treated with 250 nM RA during 10–11 hpf.** Embryos were treated with vehicle DMSO (WT), microinjected with *scl* and *lmo2* mRNA at 1–2 cell stage (*scl/lmo2* mRNA), treated with 250 nM RA during 10 to 11 hpf (250 nM RA 10–11 hpf), or microinjected with *scl* and *lmo2* mRNA at 1–2-cell stage and then treated with 250 nM RA during 10 to 11hpf (RA+*scl/lmo2* mRNA), respectively. To exclude the myeloid cells derived from ICM, embryos that were removed tails and trunks at 24 hpf were used to detect relative expression levels of myeloid markers *lcp1* and *mpx* by qRT-PCR. Overexpression of *scl* and *lmo2* did not change expression levels of *lcp1* and *mpx* in wild type embryos but significantly rescued the inhibited expressions of *lcp1* and *mpx* in the embryos treated with 250 nM during 10–11 hpf. **: P<0.01.
(TIF)

Figure S3 **Effect of excessive RA treatment during 10–11 hpf on expressions of** ***gata5*** **and** ***gata6*** **in the RA-treated embryos at 14 hpf.** All flat-mounted embryos are positioned anterior left and dorsal front. Embryos treated with vehicle DMSO (A, C) and 250 nM RA from 10–11 hpf were examined for expressions of *gata5* (A, B) and *gata6* (C, D) at 14 hpf, respectively. Expression of *myoD* in somites was used for staging and *ntl* expression was used for labeling embryonic axial mesoderm. The number shown in the lower left-hand corner of each panel is the number of embryos exhibiting the typical phenotype shown in the panel to the number of embryos totally observed. The embryos treated with 250 nM RA from 10–11 hpf exhibited similar expression of *gata5* but somehow increased expression of *gata6* in the region between the anterior ends of their expression domains and the anterior end of *ntl* expression domain; however, they displayed significantly increased *gata5* expression but greatly reduced *gata6* expression in the region between the posterior ends of their expression domains and the anterior end of *ntl* expression domain, respectively.
(TIF)

Figure S4 **The ablated primitive myelopoiesis in** ***gata4/5/6*** **-depleted embryos can be rescued by treating with 10 µM DEAB starting before 11 hpf.** All embryos were positioned anterior left and lateral front. Embryos were microinjected with *gata4*-MO and *gata6*-MO at 1–2-cell stage (B–H, J–P) and then treated with vehicle DMSO (B, J) or 10 µM DEAB continuously starting from 1–2-cell stage (C, K), 3 hpf (D, L), 5 hpf (E, M), 7 hpf (F, N), 9 hpf (G, O), and 11 hpf (H, P), respectively. The embryos were then grown together with wild type control embryos (A, I) to 24 hpf for examining expressions of myeloid markers *lcp1* (A–H) and *mpx* (I, P) respectively. The number shown in the lower left-hand corner of each panel is the number of embryos exhibiting the typical phenotype shown in the panel to the number of embryos totally observed. Treatment with DEAB before 11 hpf can well rescue the abolished primitive myelopoiesis in *gata4/5/6*-depleted embryos (C–G; K–O) whereas the treatment after 11 hpf hardly rescued the primitive myelopoiesis (H, P).
(TIF)

Figure S5 **qRT-PCR analysis shows that the increased primitive myelopoiesis due to overexpressing** ***gata4/6*** **is blocked by treating the embryos with 250 nM RA during 10–11 hpf.** Embryos were microinjected with *gata4* mRNA plus *gata6* mRNA at 1–2-cell stage. They were treated with 250 nM RA (*gata4/6* mRNA+RA) or vehicle DMSO (*gata4/6* mRNA) during 10–11 hpf and then grown with wild type embryos (WT) to 24 hpf for examining expression level changes of myeloid markers *lcp1* and *mpx* by qRT-PCR. Overexpressing *gata4/6* into wild type embryos significantly increased expressions of *lcp1* and *mpx* but the increased expressions were blocked by treating the embryos with 250 nM RA during 10–11 hpf. *: P<0.05; **: P<0.01.
(TIF)

Figure S6 **Proposed model showing the epistatic relationship of RA signaling with the genes controlling zebrafish primitive myelopoiesis.** RA signaling works downstream of *gata4*/5/6, upstream of, or parallel to *cloche*, and upstream of *scl* to control the formation of anterior hemangioblasts marked by expressions of *scl*, *lmo2*, *gata2* and *etsrp* that give rise to the primitive myeloid precursors marked by expression of *pu.1*.
(TIF)

Table S1 **Sequences of morpholinos used.**
(DOC)

Table S2 **Sequences of primers used in cloning full-length cDNAs for** ***in vitro*** **mRNA synthesis.**
(DOC)

Table S3 **Sequences of primers used in cloning cDNAs for RNA probes.**
(DOC)

Table S4 **Sequences of primers used in qRT-PCR.**
(DOC)
