# Supplementary figures and images for: Retinoic Acid Signaling Plays a Restrictive Role in Zebrafish Primitive Myelopoiesis
Source: PLoS One. 2012 Feb 17;7(2):e30865. doi: 10.1371/journal.pone.0030865 (PMC3281886; doi:10.1371/journal.pone.0030865)

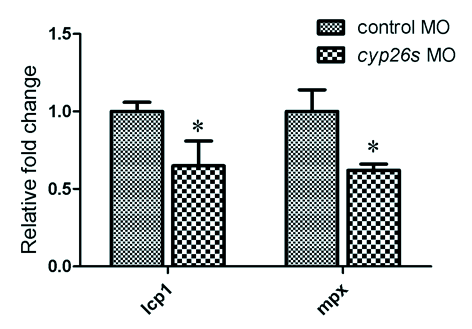

Supplement: Figure S1 — qRT-PCR analysis shows the relative expression levels of lcp1 and mpx are significantly reduced in cyp26s morphants. Embryos were microinjected with control MO (Control MO) or cyp26a1-MO plus cyp26b1-MO plus cyp26c1-MO (cyp26s MO) at 1–2-cell stage and then examined for expressions of myeloid markers lcp1 and mpx at 26 hpf by qRT-PCR. *: P<0.05. (TIF) [file pone.0030865.s001.tif]

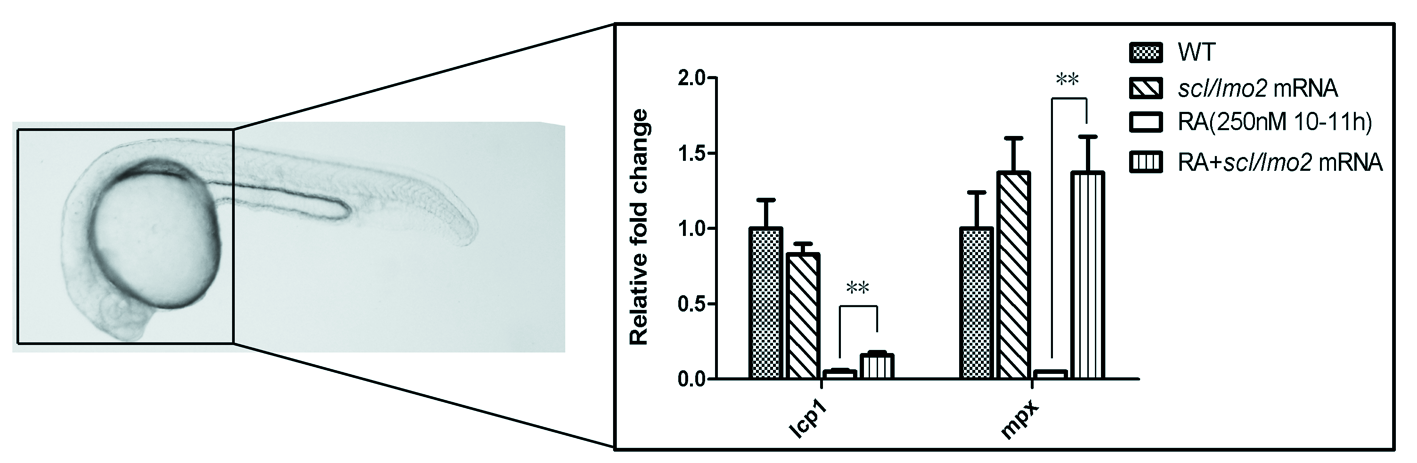

Supplement: Figure S2 — qRT-PCR analysis shows overexpression of scl and lmo2 into zebrafish embryos partially rescues the defective primitive myelopoiesis in the embryos treated with 250 nM RA during 10–11 hpf. Embryos were treated with vehicle DMSO (WT), microinjected with scl and lmo2 mRNA at 1–2 cell stage (scl/lmo2 mRNA), treated with 250 nM RA during 10 to 11 hpf (250 nM RA 10–11 hpf), or microinjected with scl and lmo2 mRNA at 1–2-cell stage and then treated with 250 nM RA during 10 to 11hpf (RA+scl/lmo2 mRNA), respectively. To exclude the myeloid cells derived from ICM, embryos that were removed tails and trunks at 24 hpf were used to detect relative expression levels of myeloid markers lcp1 and mpx by qRT-PCR. Overexpression of scl and lmo2 did not change expression levels of lcp1 and mpx in wild type embryos but significantly rescued the inhibited expressions of lcp1 and mpx in the embryos treated with 250 nM during 10–11 hpf. **: P<0.01. (TIF) [file pone.0030865.s002.tif]

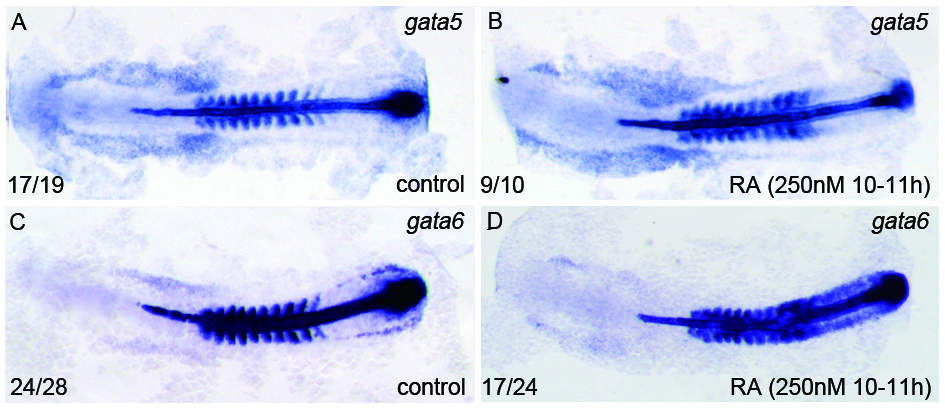

Supplement: Figure S3 — Effect of excessive RA treatment during 10–11 hpf on expressions of gata5 and gata6 in the RA-treated embryos at 14 hpf. All flat-mounted embryos are positioned anterior left and dorsal front. Embryos treated with vehicle DMSO (A, C) and 250 nM RA from 10–11 hpf were examined for expressions of gata5 (A, B) and gata6 (C, D) at 14 hpf, respectively. Expression of myoD in somites was used for staging and ntl expression was used for labeling embryonic axial mesoderm. The number shown in the lower left-hand corner of each panel is the number of embryos exhibiting the typical phenotype shown in the panel to the number of embryos totally observed. The embryos treated with 250 nM RA from 10–11 hpf exhibited similar expression of gata5 but somehow increased expression of gata6 in the region between the anterior ends of their expression domains and the anterior end of ntl expression domain; however, they displayed significantly increased gata5 expression but greatly reduced gata6 expression in the region between the posterior ends of their expression domains and the anterior end of ntl expression domain, respectively. (TIF) [file pone.0030865.s003.tif]

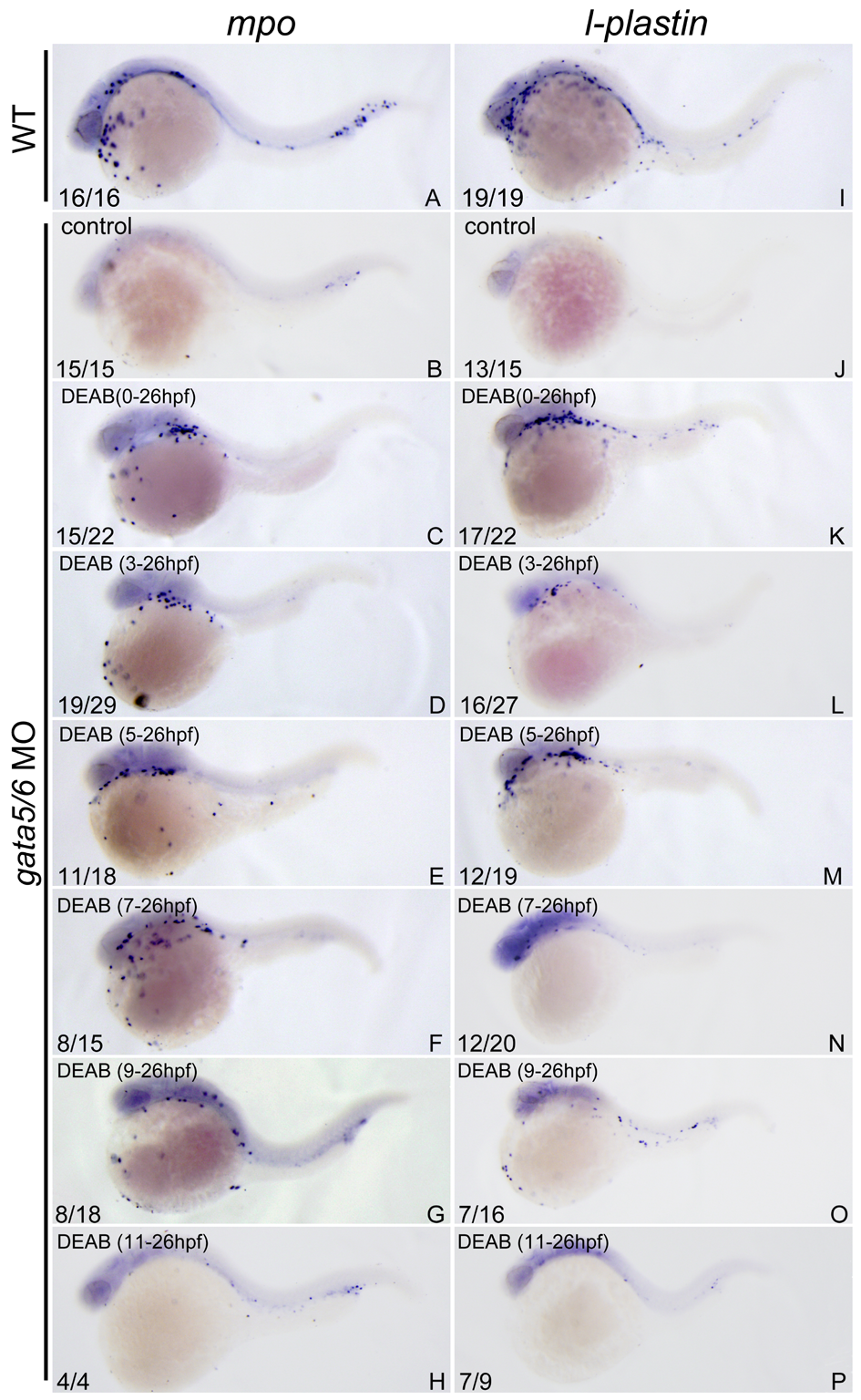

Supplement: Figure S4 — The ablated primitive myelopoiesis in gata4/5/6 -depleted embryos can be rescued by treating with 10 µM DEAB starting before 11 hpf. All embryos were positioned anterior left and lateral front. Embryos were microinjected with gata4-MO and gata6-MO at 1–2-cell stage (B–H, J–P) and then treated with vehicle DMSO (B, J) or 10 µM DEAB continuously starting from 1–2-cell stage (C, K), 3 hpf (D, L), 5 hpf (E, M), 7 hpf (F, N), 9 hpf (G, O), and 11 hpf (H, P), respectively. The embryos were then grown together with wild type control embryos (A, I) to 24 hpf for examining expressions of myeloid markers lcp1 (A–H) and mpx (I, P) respectively. The number shown in the lower left-hand corner of each panel is the number of embryos exhibiting the typical phenotype shown in the panel to the number of embryos totally observed. Treatment with DEAB before 11 hpf can well rescue the abolished primitive myelopoiesis in gata4/5/6-depleted embryos (C–G; K–O) whereas the treatment after 11 hpf hardly rescued the primitive myelopoiesis (H, P). (TIF) [file pone.0030865.s004.tif]

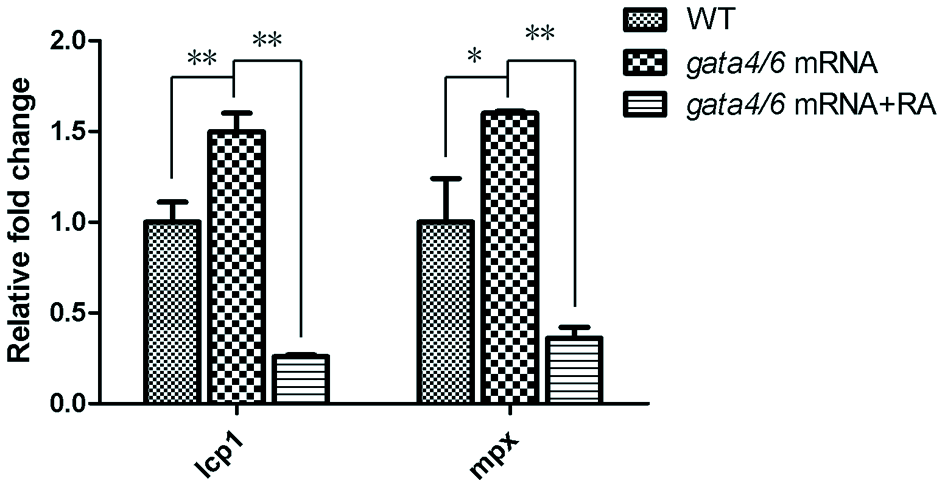

Supplement: Figure S5 — qRT-PCR analysis shows that the increased primitive myelopoiesis due to overexpressing gata4/6 is blocked by treating the embryos with 250 nM RA during 10–11 hpf. Embryos were microinjected with gata4 mRNA plus gata6 mRNA at 1–2-cell stage. They were treated with 250 nM RA (gata4/6 mRNA+RA) or vehicle DMSO (gata4/6 mRNA) during 10–11 hpf and then grown with wild type embryos (WT) to 24 hpf for examining expression level changes of myeloid markers lcp1 and mpx by qRT-PCR. Overexpressing gata4/6 into wild type embryos significantly increased expressions of lcp1 and mpx but the increased expressions were blocked by treating the embryos with 250 nM RA during 10–11 hpf. *: P<0.05; **: P<0.01. (TIF) [file pone.0030865.s005.tif]

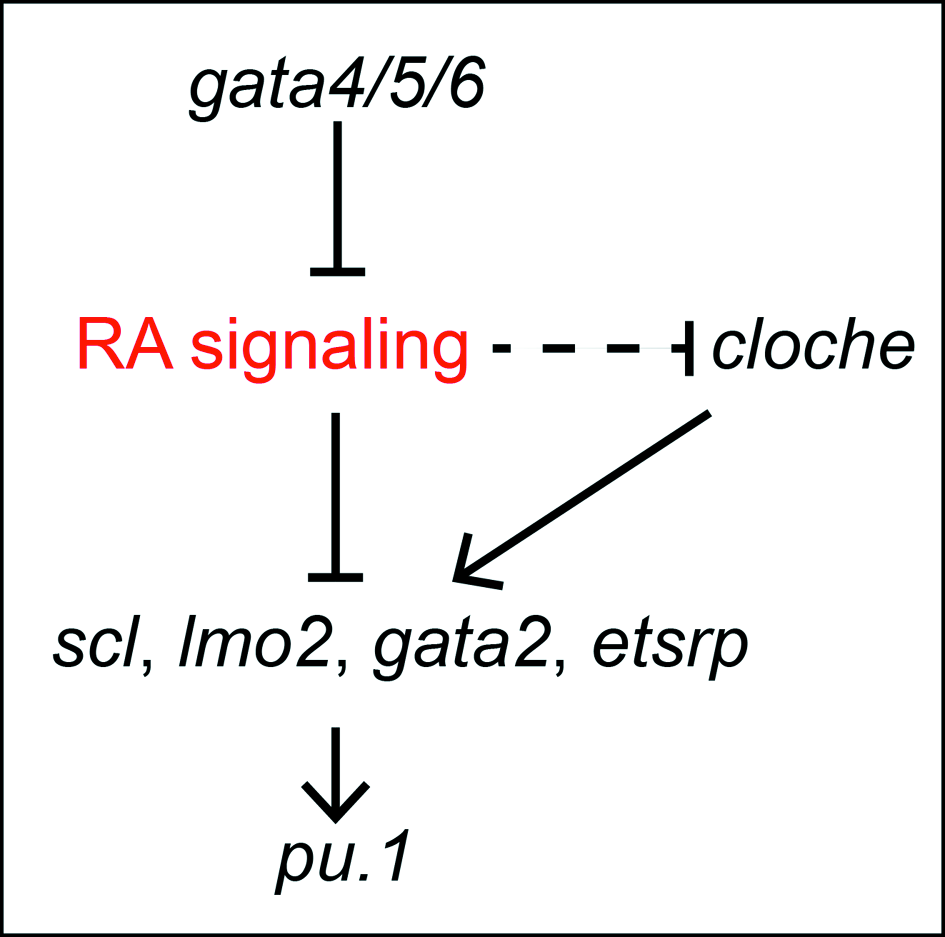

Supplement: Figure S6 — Proposed model showing the epistatic relationship of RA signaling with the genes controlling zebrafish primitive myelopoiesis. RA signaling works downstream of gata4/5/6, upstream of, or parallel to cloche, and upstream of scl to control the formation of anterior hemangioblasts marked by expressions of scl, lmo2, gata2 and etsrp that give rise to the primitive myeloid precursors marked by expression of pu.1. (TIF) [file pone.0030865.s006.tif]
