# Supplementary material for: Retinoic Acid Signaling Plays a Restrictive Role in Zebrafish Primitive Myelopoiesis
Source: PLoS One. 2012 Feb 17;7(2):e30865. doi: 10.1371/journal.pone.0030865 (PMC3281886; doi:10.1371/journal.pone.0030865)
Supplement: Table S1 — Sequences of morpholinos used. (DOC) [file pone.0030865.s007.doc]

**Table S1**. Sequences of morpholinos used.

| **Targeted gene** | **Sequence (5’-3’)** | **References*** |
| --- | --- | --- |
| control | CCT CTT ACC TCA GTT ACA ATT TAT A |  |
| *gata5* | TGT TAA GAT TTT TAC CTA TAC TGG A | 1 |
| *gata6* | AGC TGT TAT CAC CCA GGT CCA TCC A | 2 |
| *cyp26a1* | CGC GCA ACT GAT CGC CAA AAC GAA A | 3 |
| *cyp26b1* | CTC GAA GAG CAT GGC TGT GAA CGT C | 4 |
| *cyp26c1* | AAC TAC GGT TAT CCT CAC CTT GCG C | 4 |
| *lycat* | AAC ACA CAC CAC GAG GAG ACA CCA T | 5 |
| *aldh1a2* | GCA GTT CAA CTT CAC TGG AGG TCA T | 6 |

*References:

1. Peterkin T, Gibson A, Patient R (2007) Redundancy and evolution of GATA factor requirements in development of the myocardium. Dev Biol 311: 623-635.

2. Peterkin T, Gibson A, Patient R (2003) GATA-6 maintains BMP-4 and Nkx2 expression during cardiomyocyte precursor maturation. EMBO J 22: 4260-4273.

3. Emoto Y, Wada H, Okamoto H, Kudo A, Imai Y (2005) Retinoic acid-metabolizing enzyme Cyp26a1 is essential for determining territories of hindbrain and spinal cord in zebrafish. Dev Biol 278: 415-427.

4. Hernandez RE, Putzke AP, Myers JP, Margaretha L, Moens CB (2007) Cyp26 enzymes generate the retinoic acid response pattern necessary for hindbrain development. Development 134: 177-187.

5. Xiong JW, Yu Q, Zhang J, Mably JD (2008) An acyltransferase controls the generation of hematopoietic and endothelial lineages in zebrafish. Circ Res 102: 1057-1064.

6. Begemann G, Schilling TF, Rauch GJ, Geisler R, Ingham PW (2001) The zebrafish neckless mutation reveals a requirement for raldh2 in mesodermal signals that pattern the hindbrain. Development 128: 3081-3094.
