# Supplementary material for: Retinoic Acid Signaling Plays a Restrictive Role in Zebrafish Primitive Myelopoiesis
Source: PLoS One. 2012 Feb 17;7(2):e30865. doi: 10.1371/journal.pone.0030865 (PMC3281886; doi:10.1371/journal.pone.0030865)
Supplement: Table S2 — Sequences of primers used in cloning full-length cDNAs for in vitro mRNA synthesis. (DOC) [file pone.0030865.s008.doc]

**Table S2.** Sequences of primers used in cloning full-length cDNAs for *in vitro* mRNA synthesis.

| **Primer name** | **Sequence (5’-3’)** | **GenBank Accession Number** |
| --- | --- | --- |
| *scl*-F | GTA CAT TTT CTG GGA TCG CG | NM_213237 |
| *scl*-R | AGT CTG TCA GTG TCT CCA ATC | NM_213237 |
| *lmo2*-F | GAA CAG GTG CAT CTC TGA AGC G | NM_131111 |
| *lmo2*-R | GCG ACA AGC ATT TAG AGC ACA GC | NM_131111 |
| *gata4*-F | GCT CGT GGA GAA TAA TCG C | NM_131236 |
| *gata4*-R | TGC GTT TAT GCC AGA ATC AG | NM_131236 |
| *gata6*-F | CCT CAT TGT GGA CCC TAC C | NM_131557 |
| *gata6*-R | AAT TCA GCC TCA AGA TCA CC | NM_131557 |
| *hoxb5b*-F | GGC GCT GGA TTT TAA CGA CC | NM_131537 |
| *hoxb5b*-R | TGA AGG TGC AGC GCT GTA AC | NM_131537 |
