# Supplementary material for: Retinoic Acid Signaling Plays a Restrictive Role in Zebrafish Primitive Myelopoiesis
Source: PLoS One. 2012 Feb 17;7(2):e30865. doi: 10.1371/journal.pone.0030865 (PMC3281886; doi:10.1371/journal.pone.0030865)
Supplement: Table S3 — Sequences of primers used in cloning cDNAs for RNA probes. (DOC) [file pone.0030865.s009.doc]

**Table S3.** Sequences of primers used in cloning cDNAs for RNA probes.

| **Primer name** | **Sequence (5’-3’)** | **GenBank Accession Number** |
| --- | --- | --- |
| *gata5*-F | ACT AGC CTA CAA TCT GGG CG | NM_131235 |
| *gata5*-R | AGG TTT CAG TGG AGG TCT CG | NM_131235 |
| *etsrp*-F | GTG AAA TAA GGA CTC AGT G | NM_001037375 |
| *etsrp*-R | AAA GGC ACG ACG TCT GAT G | NM_001037375 |
| *gata2*-F | TTT CTA AGC GCG GAC ACT TG | NM_131233 |
| *gata2*-R | TTA TTC AGT GCC CCT GTG G | NM_131233 |
| *hand2*-F | TCC ACC TCA TTG ATT CCA CA | NM_131626 |
| *hand2*-R | CAG ATG GCC TCA TTT CGT CT | NM_131626 |
