# Supplementary material for: Retinoic Acid Signaling Plays a Restrictive Role in Zebrafish Primitive Myelopoiesis
Source: PLoS One. 2012 Feb 17;7(2):e30865. doi: 10.1371/journal.pone.0030865 (PMC3281886; doi:10.1371/journal.pone.0030865)
Supplement: Table S4 — Sequences of primers used in qRT-PCR. (DOC) [file pone.0030865.s010.doc]

**Table S4**. Sequences of primers used in qRT-PCR.

| **Primer name** | **Sequence (5’-3’)** | **References*/GenBank Accession Number** |
| --- | --- | --- |
| *l-plastin*-QF | GAA GCT CTG ATC GCT CTG CT | 1 |
| *l-plastin*-QR | GCT TCT TTT CAT CCG TCA GG | 1 |
| *mpo*-QF | GGG GCA GAA GAA GAA AGT CC | 1 |
| *mpo*-QR | CCC TTG CTA AAC TCT CAT CTC G | 1 |
| *aldh1a2*-QF | CAT TTT TGC AGA TGC TGA TTT TG | 2 |
| *aldh1a2*-QR | CAA AGA TAC GGG AAC CAG CAG T | 2 |
| *β-actin-*QF | CGA GCA GGA GAT GGG AAC C | NM_131031 |
| *β-actin-*QR | CAA CGG AAA CGC TCA TTG C | NM_131031 |

*References:

1. Ma AC, Ward AC, Liang R, Leung AY (2007) The role of jak2a in zebrafish hematopoiesis. Blood 110: 1824-1830.

2. Alsop D, Matsumoto J, Brown S, Van Der Kraak G (2008) Retinoid requirements in the reproduction of zebrafish. Gen Comp Endocrinol 156: 51-62.
